# Insights from introspection: a commentary on Gould et al. (2014), “An extended case study on the phenomenology of spatial form synaesthesia”

**DOI:** 10.3389/fnhum.2014.00439

**Published:** 2014-07-02

**Authors:** Mark C. Price

**Affiliations:** Psychology Faculty, University of BergenBergen, Norway

**Keywords:** synesthesia, sequence-space synesthesia, spatial form, mental imagery, visual imagery, introspection, automaticity

Gould et al. (2014) refreshingly devote an entire paper to investigating the detailed *experience* of a synaesthete (AB), using a rigorous qualitative method. Their paper addresses sequence-space synaesthesia, in which people associate members of sequences such as months or numbers with specific spatial locations that together create an overall spatial pattern or “form” for each sequence. I have previously argued that sequence-space synaesthesia shows strong continuity with normal voluntary visuospatial imagery (Price, 2009, 2013), and may be an extension of normal childhood development of visuospatial skills (Price and Pearson, 2013). Additionally I have suggested that behavioral evidence for the automaticity of this synaesthesia is less robust than often claimed (Price and Mattingley, 2013). Gould et al.'s data provide striking support for these claims.

Take continuity with normal visuospatial imagery:

1. *Some spatial forms seem indistinguishable from normal imagery:* e.g., AB's multiple and dynamic “spatial forms” for clock-time constitute a vivid visual imagery of clocks that would unlikely be considered synaesthetic if reported by a non-synaesthete.
2. *Spatial relationships of sequence items are only a subset of AB's total visualization experience:* e.g., Equations and graphs are visualized together with numerals on his number line; scenes of embryonic development are associated with his autobiographical timeline; his alphabet form resides within the dark, muddy atmosphere of a cave, itself part of an expansive landscape.
3. *Imagery when attending single sequence items is similar to normal alphanumeric imagery:* e.g., Individual numerals/letters are often imaged in isolation from the rest of AB's number or alphabet form, and although these images contain salient color, texture, and font, they do not seem so far removed from non-synaesthetic ability to visualize graphemes.
4. *Like normal imagery, aspects of the synaesthetic imagery can be somewhat modulated voluntarily*: e.g., AB can effortfully but unstably modify the font of single letters or numerals in a form.
5. *Like normal visual imagery, spatial forms can be mentally scanned:* e.g., AB appears to intentionally shift attention from one sequence member to another, progressively reconstructing his spatial form by activating long term memory of the relative locations of sequence members.

Next consider how voluntary attention mediates AB's experiences:

1. Attentional movement is required to activate parts of AB's number line that were not previously in focus.
2. Focal attention increases stability of AB's synaesthetic experience (just as maintenance of normal visual imagery is considered an effortful process, drawing on executive capacity).
3. AB superimposes his spatial forms flexibly on physical space, avoiding crowded parts of the external environment or choosing to place the form on a particular surface.
4. Without attention, spatial forms may not be induced at all. AB sometimes engages with letters and numbers without triggering any synaesthetic experience. Synaesthetic induction is not obligatory, but context dependent and susceptible to task interference.

Gould et al.'s data go beyond merely exemplifying previous claims. AB's distinction between physical space and the “mental room” in which his synaesthesia is experienced is perhaps not so novel as suggested by the authors, because it is pre-empted by earlier self-report surveys (Phillips, 1897; Sagiv et al., 2006) and autobiographical accounts (Tyler, 2005, pp. 34–35), but descriptions of complex interaction between AB's physical and mental spaces seem more detailed than previous accounts. They also challenge simplistic dichotomy between projector and associator synaesthesia. Moreover, the reliance of AB's synaesthesia on internally verbalizing the name of the inducer does seem a novel contribution to our knowledge (and is consistent with suggestions that spatial forms arise when visuospatial codes are adopted to help retention of sequences whose individual members are primarily learned via auditory exposure). Gould et al. stress that aspects of these experiences were previously unknown even to the synaesthete, and that the formal qualitative method of the Elicitation Interview (EI), adopted in their study, can uncover such previously hidden phenomenology. It should nevertheless be noted that more informal introspection can also reveal novel aspects of synaesthetic experience. For example, Tyler (2005, p. 36) describes how he first thought his synaesthetic color associations were limited to numbers, “but then I started running through the letters of the alphabet and realized that, indeed, they did evoke particular colors when I paid attention to this modality.” Whether the EI method is necessary for some kinds of discovery, or merely corroborates what can be revealed by more informal introspection, is therefore open to question.

In any case, it follows that introspective studies can transform synaesthetes' experiences rather than merely articulating them in neutral fashion. This violates the definitional synaesthetic criterion of consistency over time, convergent with other recent claims that synaesthetic concurrents can change over shorter and longer time scales (Simner, 2012; Meier et al., 2014). So how do the contents of synaesthetic experience change during detailed introspective exploration? Are the new experiences *de novo* creations, or shifts from preconscious to conscious representations. Alternatively, some synaesthetic experience may initially color what James (1890; Mangan, 1993) referred to as the unattended “fringe” of sensory awareness, but be brought into focal awareness via introspective dialog. Future work needs to empirically distinguish these possibilities.

An obvious question with single-case studies is the extent to which the participant is representative. Gould et al. consider AB a “normal” sequence-space synaesthete as his experiences are consistent with previous reports in the literature (although they also acknowledge that AB's introspective dialog may have modified his experiences beyond what is typical). However, normality is a questionable concept here because the character of this variety of synaesthesia seems to vary among individuals along many dimensions. These include automaticity, complexity, visual vs. spatial quality, projector-associator gradient, spatial reference frame and types of spatial transformation that can be applied (Price, 2009, 2013). For example, AB reports his spatial forms are flexibly placed into an environmental reference frame. By contrast, other synaesthetes report spatial forms that are more egocentrically defined. A thorough taxonomy for spatial forms is still missing from the field, but the type of research provided by Gould et al. helps us map the territory that needs to be included in such a taxonomy.

## Conflict of interest statement

The author declares that the research was conducted in the absence of any commercial or financial relationships that could be construed as a potential conflict of interest.
